# Artificial Intelligence–Assisted Bone Age Assessment to Improve the Accuracy and Consistency of Physicians With Different Levels of Experience

**DOI:** 10.3389/fped.2022.818061

**Published:** 2022-02-24

**Authors:** Xi Wang, Bo Zhou, Ping Gong, Ting Zhang, Yan Mo, Jie Tang, Xinmiao Shi, Jianhong Wang, Xinyu Yuan, Fengsen Bai, Lei Wang, Qi Xu, Yu Tian, Qing Ha, Chencui Huang, Yizhou Yu, Lin Wang

**Affiliations:** ^1^Department of Child Health Care, Children's Hospital, Capital Institute of Pediatrics, Beijing, China; ^2^Deepwise AI Lab, Beijing, China; ^3^Laboratory of Child Development and Nutriomics, Capital Institute of Pediatrics, Beijing, China; ^4^Radiology Department, Children's Hospital, Capital Institute of Pediatrics, Beijing, China

**Keywords:** bone age, artificial intelligence, China 05 RUS-CHN, accuracy, consistency, different levels of experience

## Abstract

**Background:**

The accuracy and consistency of bone age assessments (BAA) using standard methods can vary with physicians' level of experience.

**Methods:**

To assess the impact of information from an artificial intelligence (AI) deep learning convolutional neural network (CNN) model on BAA, specialists with different levels of experience (junior, mid-level, and senior) assessed radiographs from 316 children aged 4–18 years that had been randomly divided into two equal sets-group A and group B. Bone age (BA) was assessed independently by each specialist without additional information (group A) and with information from the model (group B). With the mean assessment of four experts as the reference standard, mean absolute error (MAE), and intraclass correlation coefficient (ICC) were calculated to evaluate accuracy and consistency. Individual assessments of 13 bones (radius, ulna, and short bones) were also compared between group A and group B with the rank-sum test.

**Results:**

The accuracies of senior, mid-level, and junior physicians were significantly better (all *P* < 0.001) with AI assistance (MAEs 0.325, 0.344, and 0.370, respectively) than without AI assistance (MAEs 0.403, 0.469, and 0.755, respectively). Moreover, for senior, mid-level, and junior physicians, consistency was significantly higher (all *P* < 0.001) with AI assistance (ICCs 0.996, 0.996, and 0.992, respectively) than without AI assistance (ICCs 0.987, 0.989, and 0.941, respectively). For all levels of experience, accuracy with AI assistance was significantly better than accuracy without AI assistance for assessments of the first and fifth proximal phalanges.

**Conclusions:**

Information from an AI model improves both the accuracy and the consistency of bone age assessments for physicians of all levels of experience. The first and fifth proximal phalanges are difficult to assess, and they should be paid more attention.

## Introduction

Bone age assessment (BAA) is a very important parameter of a child's growth assessment in clinical practice, and is widely used in pediatric endocrinology (1, 2). In China, the Greulich-Pyle atlas method (3, 4) and the Standard of Skeletal Maturity of the Hand and Wrist for Chinese (China 05 RUS-CHN method) (5) are widely used BAA methods. The Greulich-Pyle method is simple and easy to apply, but assessment results are greatly influenced by the experience of the observer (6). The China 05 RUS-CHN method, which is established with Chinese as the reference group, evaluates and scores each bone of the 14 skeletons. Thus the China 05 RUS-CHN method is complicated and time-consuming, the evaluation process requires extensive experience, and the assessment results also differ with physicians' seniority of experience (7). Therefore, there is an urgent need to establish a rapid, reliable automated BAA system (8, 9).

Recently, deep learning-based BAA systems have received attention in both medical and computer science communities (10). In the Radiological Society of North America Pediatric Bone Age Machine Learning Challenge, which used the mean Greulich-Pyle atlas reading of four human reviewers as reference standard, top teams achieved mean absolute errors (MAEs) from 4.265 to 4.907 months (11, 12). Ren X et al. (13) used a supervised convolutional neural network model and achieved an average MAE of 5.2 months for the Radiological Society of North America dataset. Meanwhile, Retrieval of an X-ray image from a picture archiving and communication system, processing the image, and reading the bone age required approximately 1.5 s, while radiologists required 1.4 to 7.9 min to assess bone age. Although the advantages of using automated BAA have been demonstrated, most studies (14, 15) only compared AI and physicians' BAA, which does not demonstrate the value of AI assistance to physicians with different levels of experience.

To the best of our knowledge, there has been no literature on the influence of physicians' levels of experience on AI-assisted BAA; therefore, we conducted a multi-level investigation to validate the impact of deep learning on the accuracy and the consistency of BAA by physicians with different levels of experience.

## Methods

### Ethics

This study was conducted with institutional review board approval at the Capital Institute of Pediatrics (NumberSHERLL2020018). All participants provided informed consent.

### Participants and Methodology

**Figure 1:**
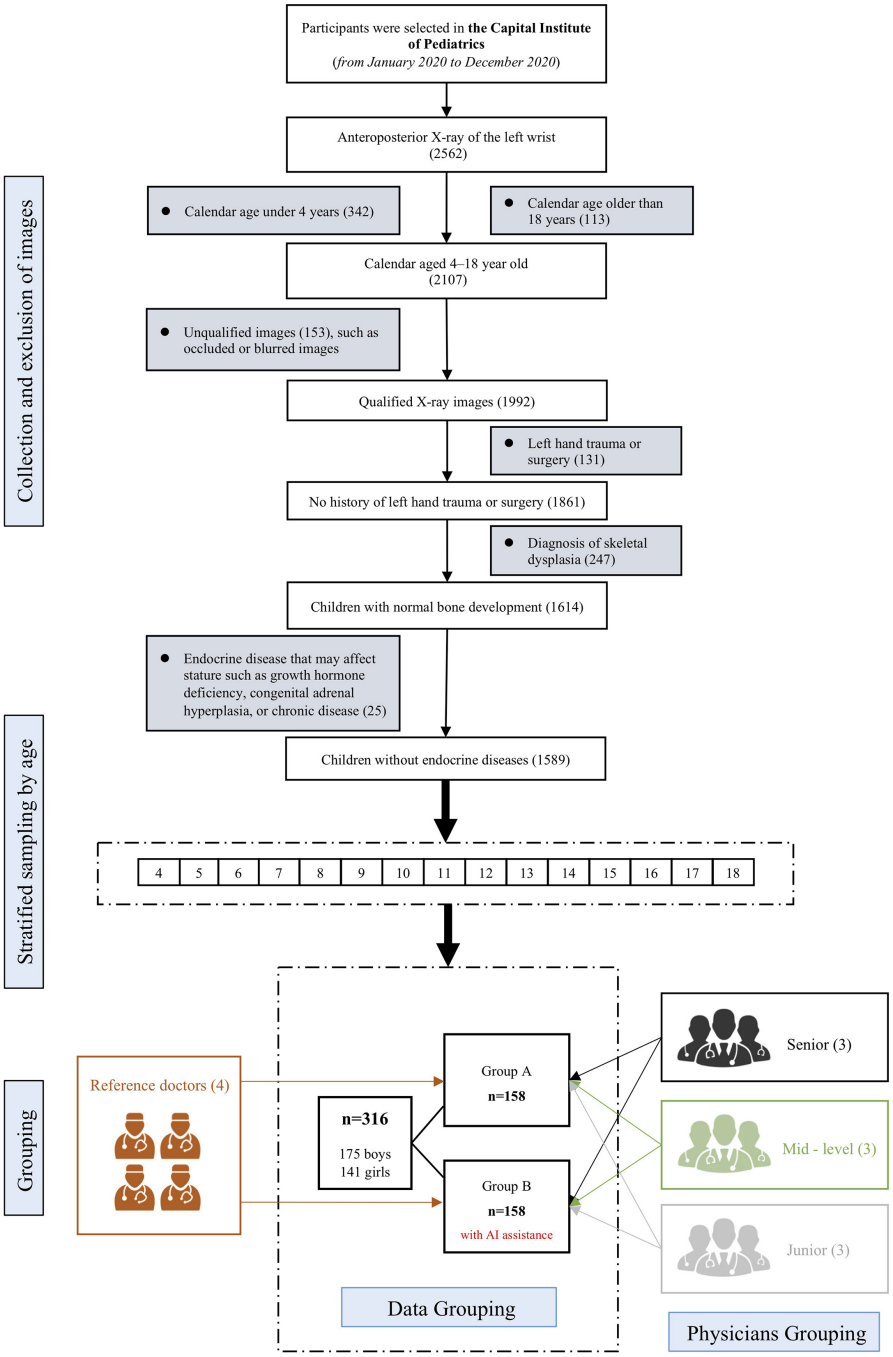
Study design flowchart. The upper part illustrates inclusion and exclusion criteria. After stratified sampling by age, an age-balanced cohort of 316 samples were extracted, which were further randomly divided into group A and group B, each with 158 samples. Both groups were independently evaluated by 4 reference standard experts and 9 physicians of different levels of experience. For group B, the 9 physicians were given AI reports before performing BAA by themselves.

Participants and methodology are illustrated in Figure 1. Participants were recruited from the Capital Institute of Pediatrics between January 2020 and December 2020. Children 4 to 18 years old who had X-rays taken for BAA were recruited. Exclusion criteria were diagnoses of skeletal dysplasia, endocrine diseases, or hereditary metabolic diseases that may affect stature (such as growth hormone deficiency, congenital adrenal hyperplasia, or chronic diseases). A total of 1,589 children were eligible. After stratification by age, 316 children were randomly selected to form two equal (*n* = 158) age-balanced cohorts (groups A and B).

Nine physicians with different levels of bone age assessment experience (three senior specialists, with more than 10 years; three mid-level specialists, with 5–10 years of experience; and three junior specialists, with less than 5 years of experience) and four experts (two radiologists, one pediatric endocrinologist, and one pediatric healthcare physician), each with more than 15 years of experience, participated in this study.

Radiographs were independently assessed, using the China 05 RUS-CHN method, by the nine physicians either with no additional information (group A) or with information from AI bone age assessment reports (group B). The physicians were blinded to others results, but were informed about each patient's sex and chronological age just as daily clinical practices. In the group B, there were three steps to evaluate the bone age. First, the physicians of group B were asked to evaluate the bone age (BA) by themselves. Second, AI's reports were given to the physicians. Third, physicians were instructed to make corrections when they double-check their BA results comparing with the AI reports. We used the average assessment of the experts, who were experienced in using the China 05 RUS-CHN method, as the reference standard. MAE was calculated as the mean of the absolute values of the difference between the physicians' assessments and the reference standard. We used MAE because it is less sensitive than root mean square error to out-of-distribution samples. Intraclass correlation coefficient (ICC) was calculated to determine the consistency of physicians' bone age assessments. Bland-Altman plots with 95% limits of agreement were used to examine bone age assessment differences between the AI model and the reference standard.

### Deep Learning Models

All radiographs were acquired using Global 1 Platform DX (General Electric). Dr. Wise Bone Age Detection and Analysis System was used as the AI model and was run on NVIDIA Graphics Processing Unit TITAN Xp. The AI models mainly consisted of a landmark detection algorithm and a bone development stage rating algorithm, as illustrated in Supplementary Figure 1. The landmark detection algorithm included two steps. Firstly, following the Faster R-CNN (16) method, hand bounding box was detected. Secondly, within the hand bounding box, target RUS bones were located using High-Resolution Net (HRNet) (17). The bone development stage rating algorithm used residual nets (ResNet34) (18) as backbone to extract epiphyseal image features of target RUS bones, which were sent to a graph convolution network module. This module combined the local image features of the epiphyseal ROI and the contextual features of adjacent epiphyseal ROIs to exploit the pattern of hand-bone growth (19).

The AI models were developed on 14,855 radiographs of different patients from six data centers in China. On an internal validation cohort of 1,486 patients, this system achieved MAE of 0.249 year (95% CI: 0.238, 0.260 years) for China 05 RUS-CHN assessments.

### Statistical Analysis

Statistical software (IBM Corp. 2013. IBM SPSS Statistics for Windows, Version 22.0. Armonk, NY; https://www.ibm.com/analytics/spss-statistics-software) and R language (https://www.r-project.org) were used for statistical analysis. We assessed the normality of continuous variables using skewness and kurtosis test. Between-group comparisons of baseline characteristics were analyzed using the chi-square test (gender), and two independent sample *t*-test (age). MAEs, ICCs and the differences for 13 bones (radius, ulna, and short bones) were compared, by physician level of experience, between assessments with and without AI assistance using the rank-sum test. Expert assessments demonstrated consistency (ICC 0.990, 95% CI: 0.987, 0.992).

*P* < 0.05 (two-sided) was considered to be statistically significant. Python 3.0 (https://www.python.org/) was used for model training and bone age calculation.

## Results

### Baseline Data

Table 1 shows that there were no statistically significant differences in age and gender (*P* > 0.05) between the children in group A and those in group B.

**Table 1:** Characteristics of children in groups A and B.

**Clinical factors**	**Group A**	**Group B**	***P*-value**
Cases	158	158	–
Calendar age (Age ± SD)	9.805 ± 3.568	9.903 ± 3.521	0.807^a^
Gender (%)			0.910^b^
female	70 (44.3%)	71 (44.9%)	
male	88 (55.7%)	87 (55.1%)	

*Data are expressed as mean (standard deviation) or count (percent). P-value was calculated by*

a *Two independent sample t-test or the*

b *Chi-squared test*.

### Model Performance

Landmarks detected by the AI model are shown in Supplementary Figure 2. The landmarks included epiphyses for the radius, the ulna, metacarpals I/III/V, proximal phalanges I/III/V, middle phalanges I/III/V, and distal phalanges I/III/V.

**Figure 2:**
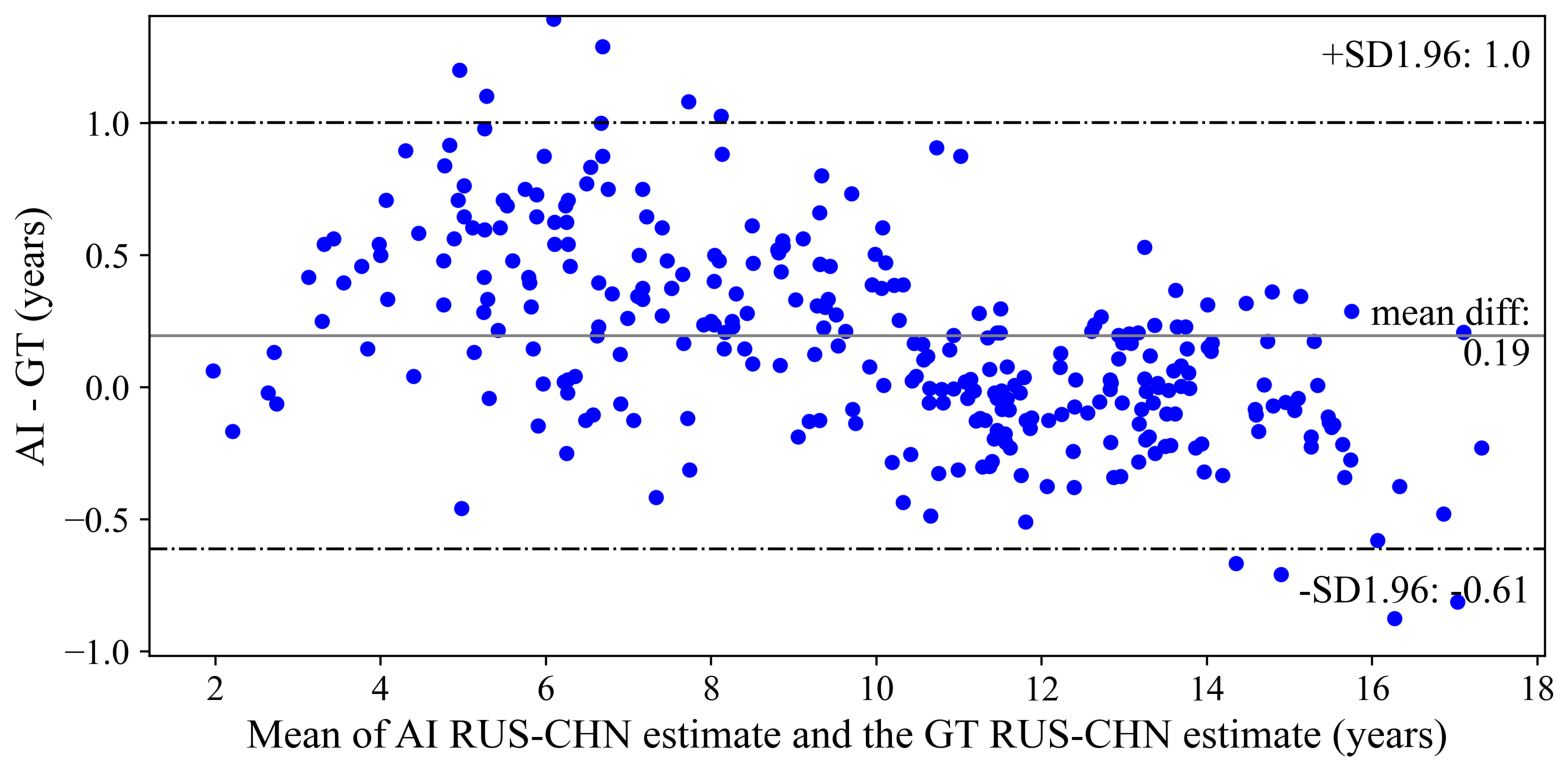
Bland-Altman plot of differences between the artificial intelligence model and reference standard bone age assessments. AI, artificial intelligence; RS, reference standard; RUS-CHN, Chinese Standard of Skeletal Maturity of the Hand and Wrist.

The Bland-Altman plot illustrating the difference between the AI model and the reference standard over the range of the mean of the two estimates are shown in Figure 2. The mean difference was +0.19 years, with 95% limits of agreement from−0.613 to +1.003 years. Qualitatively, the AI model tended to overestimate bone age for small children and underestimate bone age for older children. MAE between the AI model and reference standard was 0.332±0.312 years.

### Performance of Doctors With and Without AI Assistance

**Figure 3:**
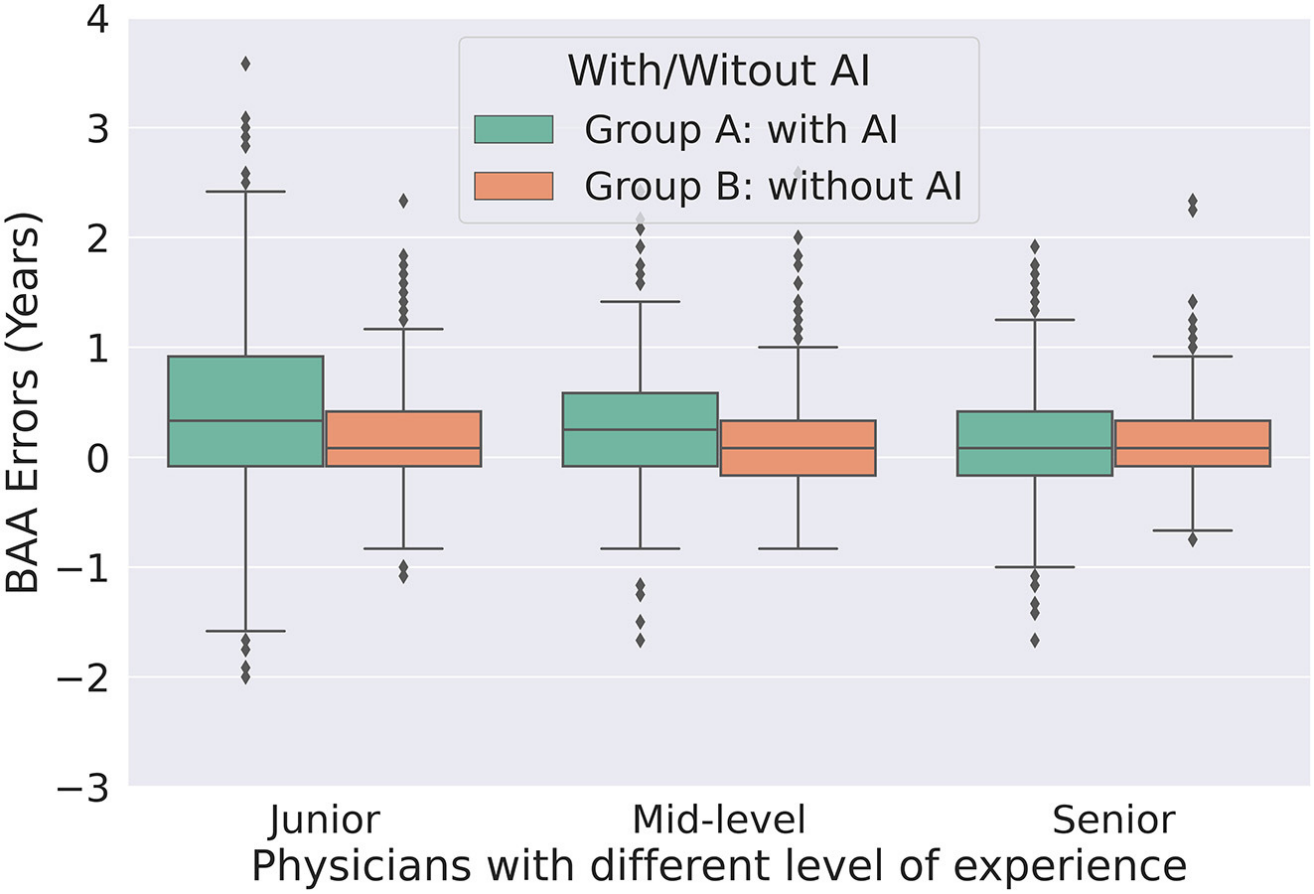
Box plot of bone age assessment errors without AI assistance (group A) and with AI model assistance (group B) for physicians of different experience. AI, artificial intelligence; BAA, bone age assessment.

The accuracies were significantly better (all *P* < 0.001) with AI assistance (MAEs 0.325, 0.344, and 0.370, respectively) than without AI assistance (MAEs 0.403, 0.469, and 0.755, respectively) for senior, mid-level, and junior physicians (Table 2). The consistency was significantly higher (all *P* < 0.001) with AI assistance (ICCs 0.996, 0.996, and 0.992, respectively) than without AI assistance (ICCs 0.987, 0.989, and 0.941, respectively) for senior, mid-level, and junior physicians (Table 2). Figure 3 utilize standard box plot diagram to show the BAA error distributions of physicians with different levels of experience. The boxes without AI assistance are filled in green, while those with AI assistance are filled in orange. The black line in the middle of the box represents the median of BAA Errors. The height of boxes, which represents the middle 50% of data points, clearly shrink with AI assistance for all three groups of physicians.

**Table 2:** Assessment performance for physicians with different levels of experience with no additional information (group A) and with artificial intelligence model assistance (group B).

**Groups**		**Mean Absolute Error (*MAE*)**	**ICC (95%CI)**
Senior physicians	group A	0.403 ± 0.368	0.987 (0.983, 0.99)
	group B	0.325 ± 0.326	0.996 (0.995, 0.997)
	*P-*value	<0.001	<0.001
Mid-level physicians	group A	0.469 ± 0.415	0.989 (0.9786, 0.992)
	group B	0.344 ± 0.356	0.996(0.995, 0.997)
	*P*-value	<0.001	<0.001
Junior physicians	group A	0.755 ± 0.679	0.941 (0.91, 0.96)
	group B	0.370 ± 0.365	0.992 (0.989, 0.994)
	*P*-value	<0.001	<0.001

*Data are expressed as mean (standard deviation). P-value was calculated by the rank-sum test or the Chi-squared test where appropriate. ICC, intraclass correlation coefficient. 95% CI, 95% confidence interval*.

Table 3 shows MAEs for assessments of 13 bones (including the radius, the ulna, and short bones). For almost every bone, MAEs for assessments with AI assistance (group B) were better than those without AI assistance (group A). For senior specialists, MAEs for the first and fifth proximal phalanges were significantly lower with AI assistance than those without (*P* < 0.05). For mid-level specialists, in addition to those for the first and fifth proximal phalanges, MAEs for the radius, the ulna, the third proximal phalanx, and the first distal phalanx were significantly lower with AI assistance than those without. For junior specialists, MAEs for all 13 bones were significantly lower with AI assistance than those without.

**Table 3:** MAE for assessments of 13 bones (radius, ulna, and short bones).

	**13 Bones**	**Mean Absolute Error (** ***MAE*** **)**		***P*-value**
		**group A**	**group B**	
Senior physicians	radius	0.403 ± 0.46	0.347 ± 0.367	0.224
	ulna	0.371 ± 0.571	0.289 ± 0.386	0.331
	metacarpal I	0.266 ± 0.356	0.265 ± 0.334	0.839
	metacarpal III	0.313 ± 0.375	0.327 ± 0.348	0.174
	metacarpal V	0.361 ± 0.423	0.281 ± 0.298	0.198
	proximal phalanx I	0.31 ± 0.332	0.258 ± 0.302	0.021
	proximal phalanx III	0.305 ± 0.357	0.271 ± 0.321	0.179
	proximal phalanx V	0.302 ± 0.369	0.233 ± 0.315	0.004
	middle phalanx III	0.291 ± 0.358	0.262 ± 0.333	0.177
	middle phalanx V	0.792 ± 1.37	1.009 ± 1.843	0.398
	distal phalanx I	0.353 ± 0.556	0.344 ± 0.594	0.954
	distal phalanx III	0.319 ± 0.415	0.291 ± 0.283	0.383
	distal phalanx V	0.301 ± 0.382	0.277 ± 0.306	0.927
Mid-level physicians	radius	0.468 ± 0.464	0.368 ± 0.375	0.004
	ulna	0.373 ± 0.498	0.288 ± 0.389	0.045
	metacarpal I	0.354 ± 0.452	0.292 ± 0.345	0.329
	metacarpal III	0.411 ± 0.465	0.334 ± 0.373	0.112
	metacarpal V	0.335 ± 0.396	0.297 ± 0.317	0.855
	proximal phalanx I	0.36 ± 0.388	0.281 ± 0.304	0.006
	proximal phalanx III	0.38 ± 0.436	0.291 ± 0.343	0.004
	proximal phalanx V	0.32 ± 0.382	0.251 ± 0.318	0.008
	middle phalanx III	0.32 ± 0.394	0.291 ± 0.365	0.231
	middle phalanx V	1.03 ± 1.888	0.951 ± 1.858	0.429
	distal phalanx I	0.424 ± 0.594	0.365 ± 0.799	0.020
	distal phalanx III	0.347 ± 0.459	0.31 ± 0.33	0.347
	distal phalanx V	0.377 ± 0.441	0.296 ± 0.327	0.073
Junior physicians	radius	0.765 ± 0.781	0.396 ± 0.409	<0.001
	ulna	0.786 ± 1.094	0.313 ± 0.449	<0.001
	metacarpal I	0.499 ± 0.536	0.308 ± 0.385	<0.001
	metacarpal III	0.483 ± 0.505	0.343 ± 0.361	<0.001
	metacarpal V	0.475 ± 0.511	0.342 ± 0.358	0.002
	proximal phalanx I	0.485 ± 0.504	0.312 ± 0.341	<0.001
	proximal phalanx III	0.514 ± 0.498	0.293 ± 0.362	<0.001
	proximal phalanx V	0.516 ± 0.505	0.289 ± 0.362	<0.001
	middle phalanx III	0.476 ± 0.535	0.31 ± 0.385	<0.001
	middle phalanx V	1.29 ± 1.895	1.071 ± 1.85	<0.001
	distal phalanx I	0.61 ± 0.704	0.409 ± 0.679	<0.001
	distal phalanx III	0.516 ± 0.542	0.368 ± 0.378	0.001
	distal phalanx V	0.598 ± 0.572	0.388 ± 0.412	<0.001

*Data are expressed as mean (standard deviation). P-value was calculated by the rank-sum test*.

*MAE, Mean absolute error*.

## Discussion

In this study, we compared the bone age assessment results of specialists with different levels of experience with and without AI assistance among 316 children, using China 05 RUS-CHN method. The key finding of this study was that AI assistance improved bone age assessments (decreased MAE and increased ICC) performed by specialists with different levels of experience. In particular, bone age assessments of the first and fifth proximal phalanges significantly improved with AI assistance for senior, mid-level, and junior specialists. To the best of our knowledge, this is the first cross-sectional study to explore the auxiliary diagnostic value of AI bone age assessment for specialists with different levels of experience.

We chose to use the normalized mean assessment value from four expert pediatric specialists' clinical interpretations of bone radiographs as the reference standard to minimize inherent variability. And the four specialists were engaged in radiology, child health care, growth and development, and endocrinology, to enable a stable assessment of bone age assessment intrinsic variation. Our study showed the ICC of the four experts was 0.990 (95% CI: 0.987, 0.992), therefore the reference standard can be regarded as accurate and valuable.

Several studies have compared radiological bone age determination using the Greulich-Pyle method with automated bone age assessments (14, 20–22) and have found that AI bone age assessments are comparable to human assessments (23). In a machine learning challenge (11, 12), AI bone age assessments differed from the reference standard by only 4.3 months, compared with 7.3 months for radiologists. However, it is unlikely that AI models will ever be used without radiologist input, because they are incapable of rejecting radiographs with subtle abnormalities (abnormal morphology or texture). In actual clinical application, AI results need to be reviewed by a physician. Thus, AI-assisted bone age assessment is more likely to be used in clinical applications. Yet, most previous studies emphasize the accuracy and efficiency of AI bone age assessment compared with manual results (14, 19, 23). Available literature on AI-assisted bone age assessment is scarce (24). Therefore, we compared bone age assessment by specialists with different levels of experience with and without.

## AI-Assisted Bone Age Assessment

Our findings were consistent with those of a previous study (24) that also compared the bone age assessments of physicians, using radiographs, with and without AI assistance and showed that MAEs were significantly improved in physicians with AI assistance. Moreover our study showed that MAEs were significantly improved in senior, mid-level, and junior physicians. Importantly, we found that the improvement of junior physicians was the most notable. A possible reason for this finding is that bone age classification is very meticulous work, and bone age is difficult to judge. Junior physicians typically require several years of experience with the assistance of senior doctors before they can evaluate bone age independently. Additionally, despite the statistically significance of the MAEs in senior and mid-level physicians, the actual MAEs which improved in senior and mid-level physicians with AI assistance is very low (about 1 month). Our results suggest that AI bone age assessment can assist physicians with low levels of experience. Consistency significantly increased in senior, mid-level, and junior physicians. Improvements in consistency would facilitate the adoption of the bone age report by different doctors and follow-up of pediatric patients' bone age assessments, which typically requires repeating assessments of earlier radiographs in many departments.

Another key finding was that AI assistance decreased MAE for specific bones, the first and fifth proximal phalanges, among bone age assessments performed by physicians of all levels of experience including senior, mid-level, and junior physicians. Similar results were reported by Xue-Lian Zhou and colleagues (25): human interpretations of particular bones, male capitate, hamate, the first distal and fifth middle phalanx and female capitate, the trapezoid, and the third and fifth middle phalanx, were the most inconsistent. This is likely because the China 05 RUS-CHN method is subjective—there is no standard regarding which bone should be weighted or relied upon more during the assessment (8). As for senior specialists, only the MAEs for the first and fifth proximal phalanges were significantly different between the two groups, while other 11 bones were no differences. Our results indicate that bone age assessments of the first and fifth proximal phalanges may be difficult, and they should be paid more attention during bone ageassessment.

Limitations of this study are cross-sectional study and use of single-center data; therefore, the sample size was relatively small. In addition, differences in the bone age development of children in different regions of China and the influence of different digital radiography acquisition parameters on the accuracy of AI bone age interpretation were not discussed herein. In the future, more in-depth research such as a multicenter study should be carried out to address these limitations.

In summary, AI assistance increases the accuracy and the consistency of bone age assessments performed by physicians with different levels of experience. In particular, bone age, when assessment relies upon radiographs of the first and fifth proximal phalanges, is easily misjudged.

## Data Availability Statement

The raw data supporting the conclusions of this article will be made available by the authors, without undue reservation.

## Ethics Statement

The studies involving human participants were reviewed and approved by Capital Institute of Pediatrics. Written informed consent to participate in this study was provided by the participants' legal guardian/next of kin.

## Author Contributions

LiW had the idea for and designed the study and had full access to all of the data in the study and took responsibility for the integrity of the data and the accuracy of the data analysis. XW, BZ, PG, YM, and LiW drafted the paper. BZ, PG, and YM did the data analysis. XS, TZ, JW, LeW, QX, YT, YY, and CH contributed to data acquisition. All authors critically revised the manuscript for important intellectual content and gave final approval for the version to be published. All authors agree to be accountable for all aspects of the work in ensuring that questions related to the accuracy or integrity of any part of the work are appropriately investigated and resolved.

## Funding

This research was supported by Public service development and reform pilot project, Beijing Municipal Health Commission (BMR2019-11), Capital's Funds for Health Improvement and Research (2020-2-2104), and Research Foundation of Capital Institute of Pediatrics (CXYJ-2021-08, QN-2020-09).

## Conflict of Interest

The authors declare that the research was conducted in the absence of any commercial or financial relationships that could be construed as a potential conflict of interest.

## Publisher's Note

All claims expressed in this article are solely those of the authors and do not necessarily represent those of their affiliated organizations, or those of the publisher, the editors and the reviewers. Any product that may be evaluated in this article, or claim that may be made by its manufacturer, is not guaranteed or endorsed by the publisher.
