# Supplementary material for: Artificial Intelligence–Assisted Bone Age Assessment to Improve the Accuracy and Consistency of Physicians With Different Levels of Experience
Source: Front Pediatr. 2022 Feb 24;10:818061. doi: 10.3389/fped.2022.818061 (PMC8908427; doi:10.3389/fped.2022.818061)

**Supplementary Figure 1:** Deep learning model block diagram.

Abbreviations: CH 05 RUS-CHN, Chinese Standard of Skeletal Maturity of the Hand and Wrist; HRNet, High Resolution Nets; ResNet, Residual Nets.


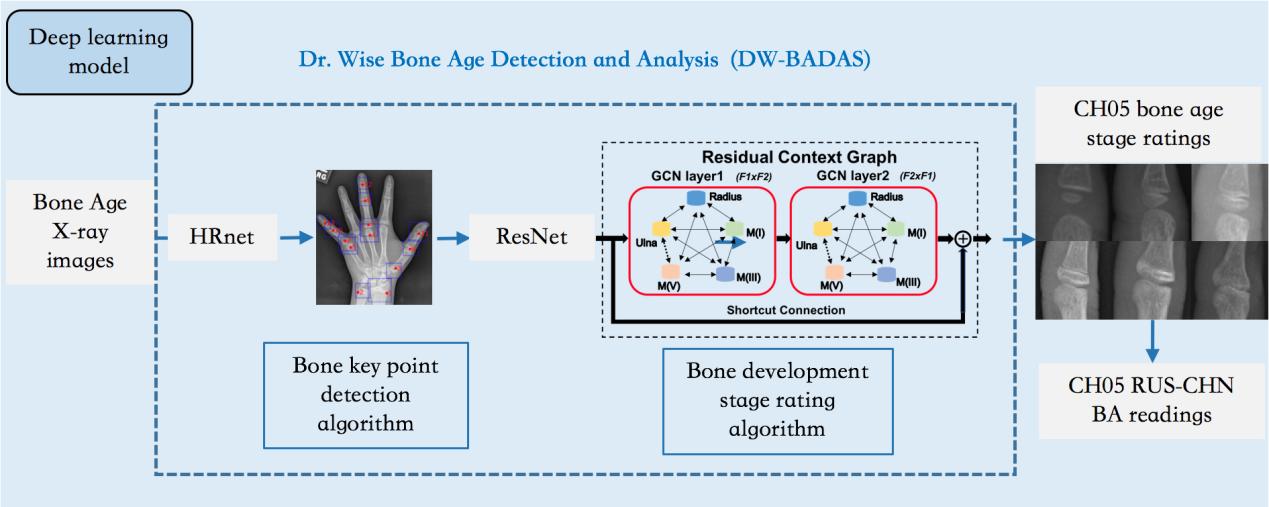


**Supplementary Figure 2:** Heatmaps showing landmarks detected by the deep learning model for three children. Columns 2-5 depict different epiphyses. Note only the fifth finger is shown for each child, for the sake of brevity. The three rows correspond to (1) standard hand position; (2) clockwise-rotation hand position; and (3) counterclockwise-rotation hand position.


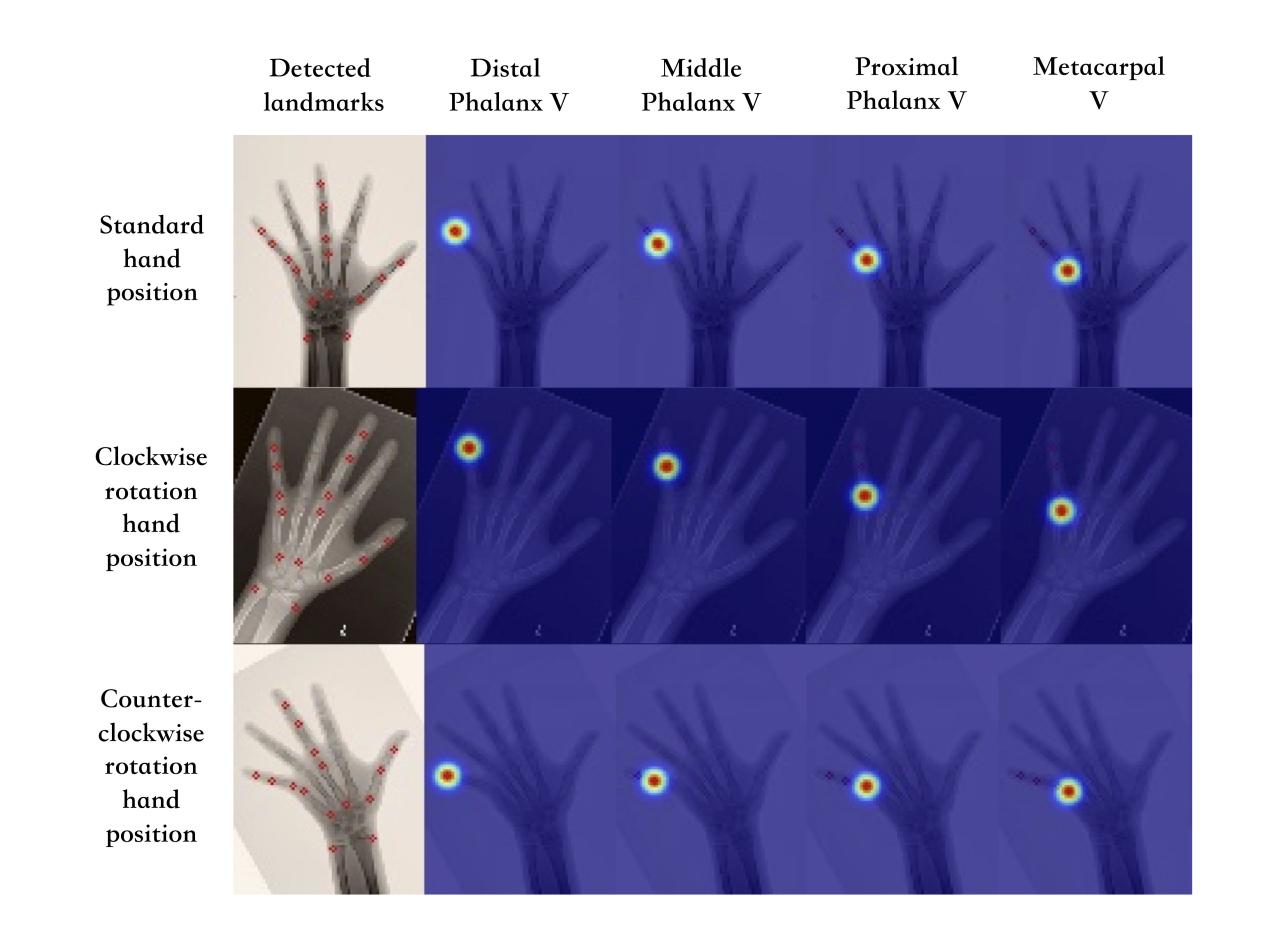


**Supplementary Figure 3:** Bland-Altman plot of BAA errors for the senior group without AI assistance.


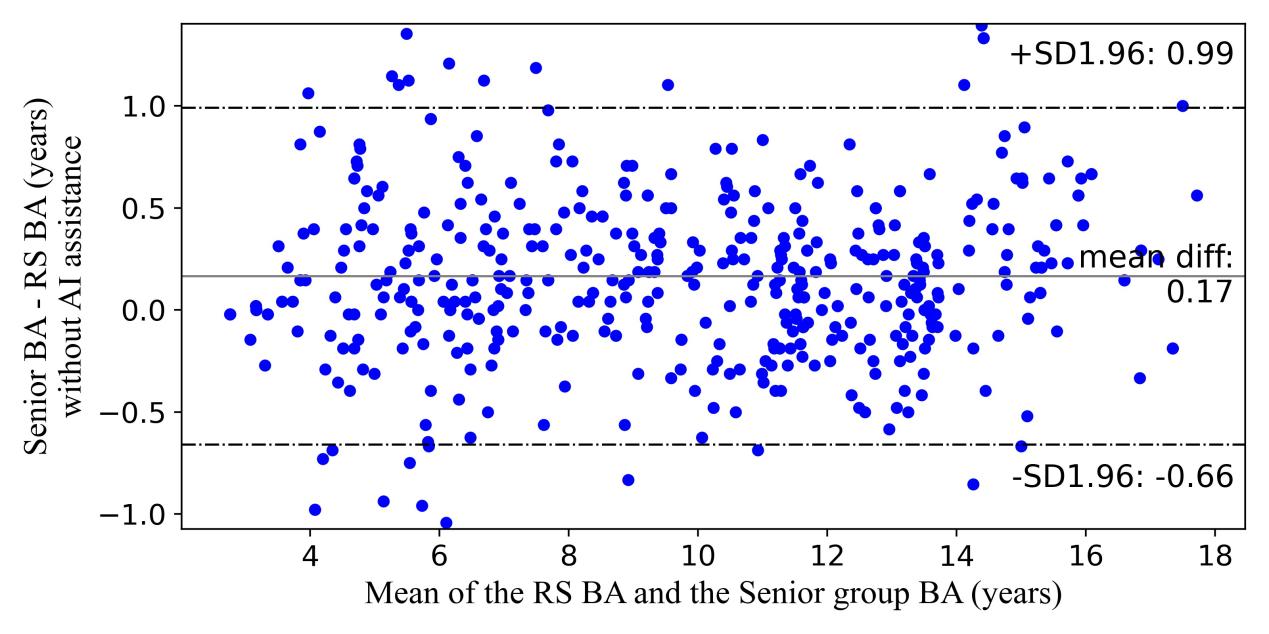


**Supplementary Figure 4:** Bland-Altman plot of BAA errors for the senior group with AI assistance.


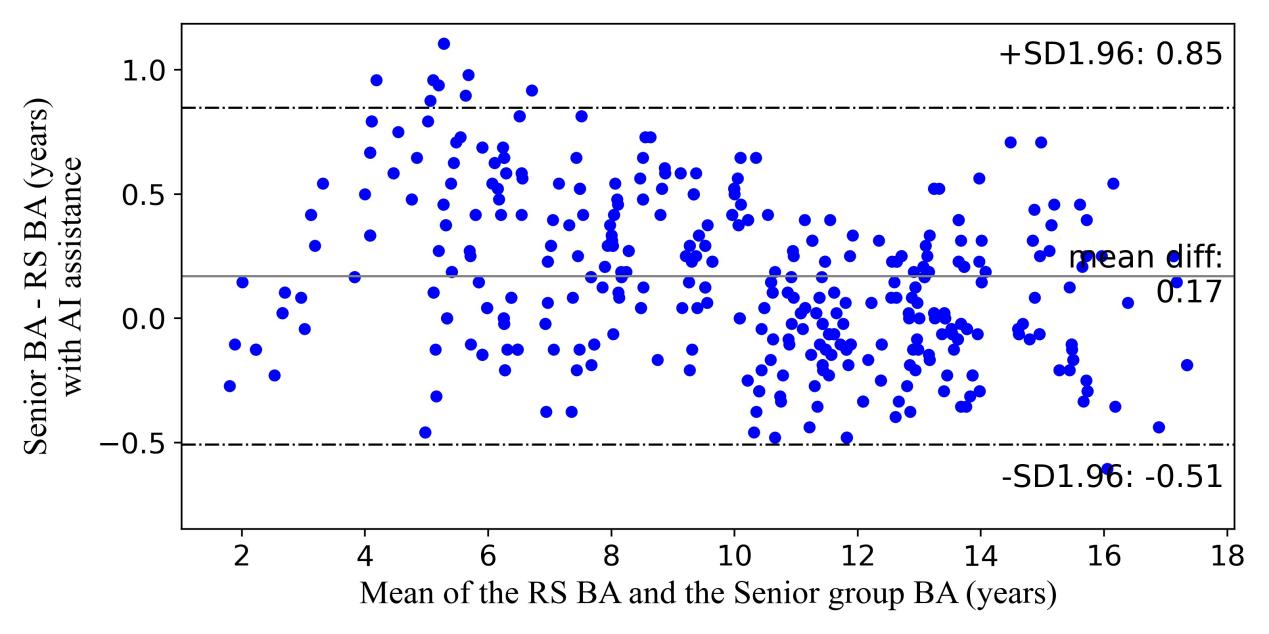


**Supplementary Figure 5:** Bland-Altman plot of BAA errors for the mid-level group without AI assistance.


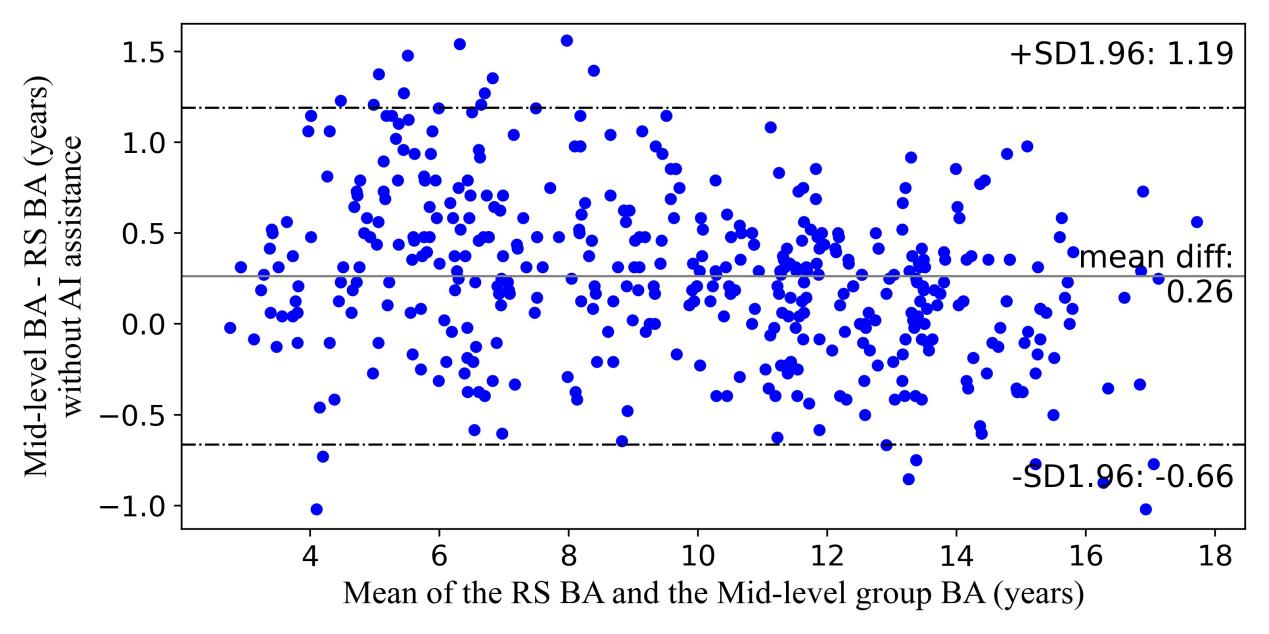


**Supplementary Figure 6:** Bland-Altman plot of BAA errors for the mid-level group with AI assistance.


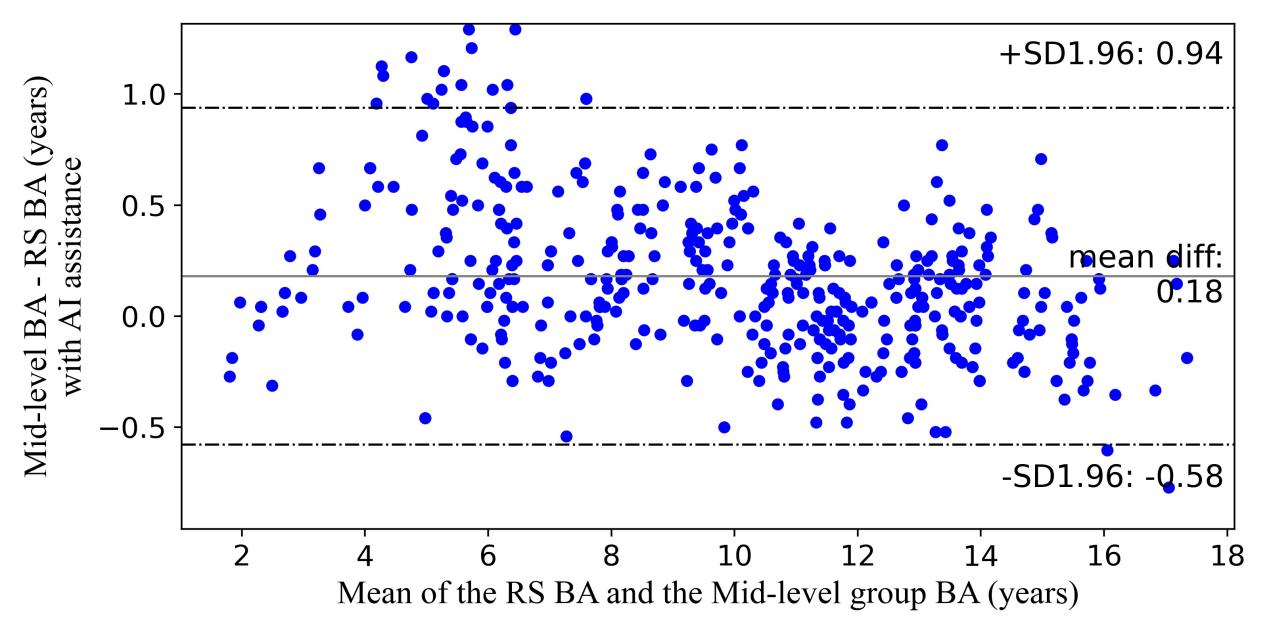


**Supplementary Figure 7:** Bland-Altman plot of BAA errors for the junior group without AI assistance.


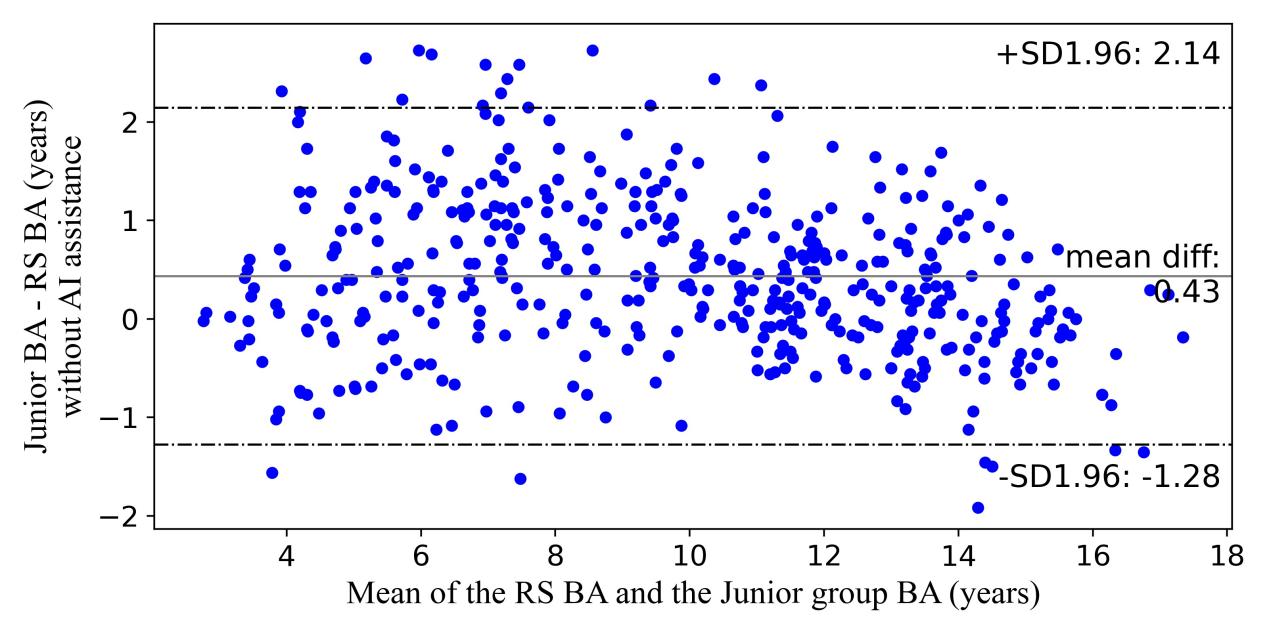


**Supplementary Figure 8:** Bland-Altman plot of BAA errors for the junior group with AI assistance.


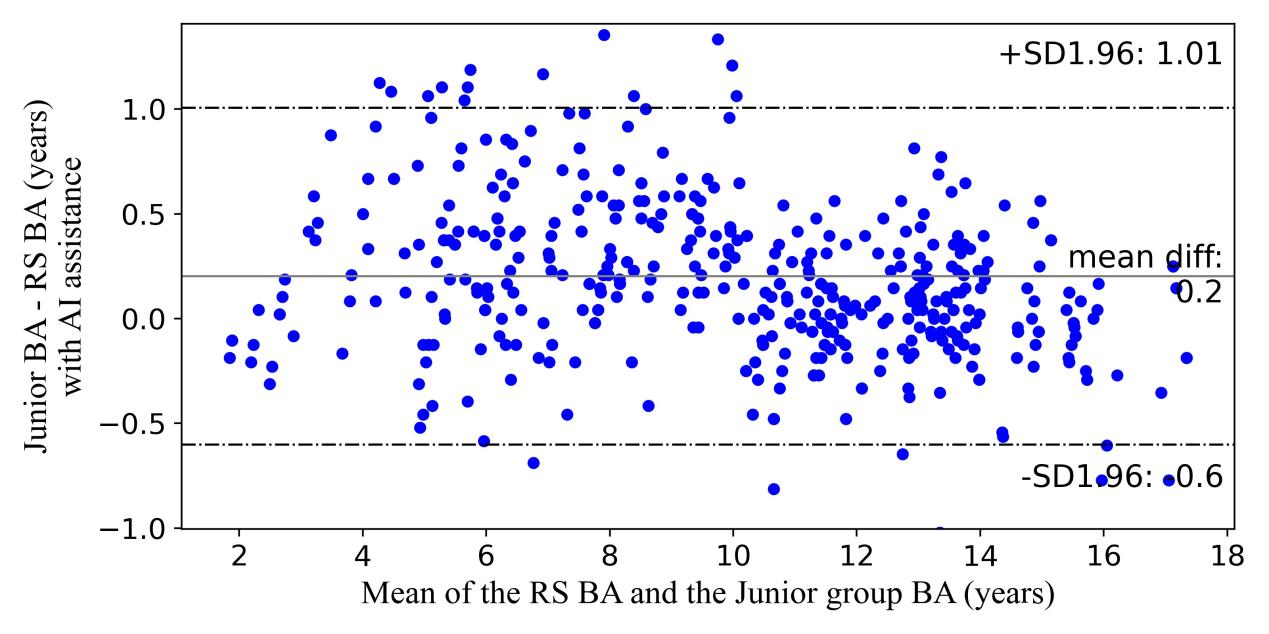

Supplement: Supplementary file 1 [file Table_1.DOCX]
